# Pharmacogenetics Approach for Personalized Tacrolimus Dosing in Heart Transplantation: A Case Report and Literature Review

**DOI:** 10.3390/genes16091010

**Published:** 2025-08-26

**Authors:** Nives Nikpalj, Jure Samardžić, Nada Božina, Livija Šimičević, Lana Ganoci, Tamara Božina

**Affiliations:** 1 General Hospital Zadar, 23000 Zadar, Croatia; nivesnikpalj@gmail.com; 2 Department of Cardiovascular Diseases, University Hospital Centre Zagreb, 10000 Zagreb, Croatia; jure.samardzic@kbc-zagreb.hr; 3 Department of Cardiovascular Diseases, School of Medicine, University of Zagreb, 10000 Zagreb, Croatia; 4 Department of Pharmacology, School of Medicine, University of Zagreb, 10000 Zagreb, Croatia; nada.bozina@mef.hr (N.B.); lana.ganoci@gmail.com (L.G.); 5 Division of Pharmacogenomics and Therapy Individualization, Department of Laboratory Diagnostics, University Hospital Centre Zagreb, 10000 Zagreb, Croatia; lsimicev@kbc-zagreb.hr; 6 Department of Chemistry and Biochemistry, School of Medicine, University of Zagreb, 10000 Zagreb, Croatia

**Keywords:** tacrolimus, pharmacogenetics, CYP3A5, CYP3A4, heart transplantation, genotype-guided dosing, drug–drug interactions, personalized medicine

## Abstract

**Background:** Tacrolimus is a cornerstone of immunosuppressive therapy following heart transplantation. Despite routine therapeutic drug monitoring (TDM), substantial interindividual variability in tacrolimus pharmacokinetics presents a persistent challenge. Pharmacogenetic profiling—particularly of *CYP3A5* and *CYP3A4* polymorphisms—offers a promising approach to individualize tacrolimus dosing and improve clinical outcomes. **Case Presentation:** We describe a 54-year-old male heart transplant recipient with persistently subtherapeutic tacrolimus trough concentrations despite escalating standard doses. Tacrolimus dosing initially started at 3.5 mg twice daily, escalated to 7.0 mg twice daily, with final maintenance dosing at 6.5 mg twice daily. TDM values were persistently subtherapeutic at 3–5 ng/mL for over a month before achieving therapeutic targets >10 ng/mL. Pharmacogenetic testing revealed a CYP3A5 expresser genotype (*1/*3) and normal CYP3A4 activity (*1/*1), suggesting enhanced metabolic clearance. In accordance with CPIC guidelines, tacrolimus dosing was intensified and supported by co-administration of diltiazem (60 mg twice daily, later adjusted to 90 mg twice daily), a CYP3A4 inhibitor. Subsequent TDM confirmed achievement of therapeutic levels. At nine months post-transplant, the patient exhibited stable graft function and excellent clinical status. **Discussion:** This case underscores the value of genotype-informed tacrolimus dosing in clinical scenarios where standard TDM is insufficient. Pharmacogenetic variation—particularly involving CYP3A5 expression—has been consistently associated with altered tacrolimus exposure and dose requirements. The literature supports routine genotyping in solid organ transplant recipients, although implementation remains limited. Additional considerations include drug–drug interactions, notably with CYP3A-modulating agents such as diltiazem and antifungals, which may further influence tacrolimus pharmacokinetics. Current evidence suggests that the utility of *CYP3A4* genotyping may be phase-dependent, being more impactful during early post-transplant periods. **Conclusions:** Incorporating pharmacogenetic data alongside TDM facilitates more precise and individualized tacrolimus therapy, optimizing immunosuppressive efficacy and minimizing risk. This case, supported by literature review, advocates for broader integration of genotype-guided strategies in transplant pharmacotherapy.

## 1. Introduction

Tacrolimus is a cornerstone of modern immunosuppressive regimens after heart transplantation [1]. Optimizing its use is crucial to increase effectiveness and reduce potential harm. The use of therapeutic drug monitoring (TDM) for tacrolimus is well established in standard clinical practice [2].

Although pharmacogenetic testing for enzymes and transporters involved in tacrolimus metabolism, particularly CYP3A5, is supported by guidelines such as those from the Clinical Pharmacogenetics Implementation Consortium (CPIC) and Dutch Pharmacogenetics Working Group (DPWG) [3,4], its routine implementation in clinical practice remains a matter of ongoing debate.

The CPIC guidelines provide strong recommendations for CYP3A5 genotype-guided tacrolimus dosing, advocating for a 1.5–2 times increase in starting dose for CYP3A5 expressers (intermediate and normal metabolizers), with a maximum dose not exceeding 0.3 mg/kg/day, followed by therapeutic drug monitoring adjustments [3]. Similarly, the DPWG recommends a 1.5-fold dose increase for heterozygous expressers and up to 2.5-fold increase for homozygous expressers, also with a maximum of 0.3 mg/kg/day [5]. Both guidelines emphasize that CYP3A5 expressers typically require substantially higher doses to achieve target trough concentrations (commonly 10–15 ng/mL in early post-transplant period), with studies showing that non-expressers achieve 1.8–2.5 times higher dose-adjusted trough concentrations compared to expressers [3,5].

The clinical relevance of *CYP3A5* genotyping is underscored by population frequency data, with current CPIC/PharmGKB data indicating that CYP3A5 expressers represent approximately 14.2% of individuals of European descent, while the majority (85.7%) are poor metabolizers (non-expressers) [6]. This population distribution highlights why many European patients achieve therapeutic tacrolimus levels with standard dosing, while a significant minority requires dose intensification.

An important pharmacogenetic consideration is the linkage disequilibrium (LD) between the CYP3A4*1B and CYP3A5*1 alleles. While CYP3A5*1 is a well-established determinant of tacrolimus metabolism and is included in current dosing guidelines, the clinical relevance of CYP3A4*1B remains uncertain. Some studies suggest that CYP3A4*1B affects tacrolimus dose requirements and tacrolimus trough concentrations, but further validation in larger and ethnically diverse cohorts is needed [3,4,7,8].

The present case highlights the clinical relevance of pharmacogenetic profiling in post-transplant immunosuppressive management, demonstrating how such profiling can clarify unexpected variability in drug levels and support a more personalized and effective therapeutic approach.

The literature review in this study highlights key findings and clinical scenarios relevant to the development of future guidelines. It also explores reasons for the limited adoption of routine pharmacogenetic testing and emphasizes the need to consider additional factors, such as drug–drug interactions, that influence tacrolimus dosing. The integration of genetic and clinical variables into transplant pharmacotherapy through patient-specific genetic profiling constitutes a more effective and safer approach to immunosuppressive treatment, with the potential to reduce intensive care unit stays and enhance cost effectiveness.

## 2. Methods of Literature Review and Case Report

For this narrative review, electronic databases PubMed (https://pubmed.ncbi.nlm.nih.gov) and PharmGKB (now ClinPGx; https://www.clinpgx.org) were searched. The search strategy covered the period from 1 January 2015 to 1 June 2025, using relevant keywords such as heart transplantation, immunosuppressive therapy, tacrolimus, cyclosporine, and pharmacogenetic markers including CYP3A4 and CYP3A5 polymorphisms. Only peer-reviewed articles published in English were included. Conference abstracts, animal studies, and reports lacking clear pharmacogenetic relevance were excluded. After identifying potential manuscripts, all authors independently reviewed and assessed their relevance, reaching consensus on inclusion. Relevant publications were narratively synthesized and organized into three thematic domains: pharmacogenetic determinants, drug–drug interactions, and cost-effectiveness considerations. Limitations of this review include its non-systematic design, restriction to English-language publications, focus on recent studies, and heterogeneity in study designs and sample sizes.

The case report was based on anonymized clinical, laboratory, and pharmacogenetic data from a 54-year-old heart transplant recipient treated at the University Hospital Centre Zagreb, School of Medicine, University of Zagreb. Data were obtained from the patient’s medical records and from the prospective study “Pharmacogenomics in Prediction of Cardiovascular Drugs Adverse Reactions” (ClinicalTrials.gov identifier NCT05307718), conducted in accordance with the Declaration of Helsinki and approved by the relevant Ethics Committees. Written informed consent was obtained from the patient.

### 2.1. Case Presentation

This case describes a 54-year-old male patient with end-stage heart failure secondary to longstanding ischemic heart disease, admitted to the University Hospital Centre Zagreb (Croatia) for further evaluation and management. The presented case is a part of a larger prospective study (“Pharmacogenomics in Prediction of Cardiovascular Drugs Adverse Reactions”) that started on 1 January 2022, and will last 60 months and include 1200 subjects. The study (ClinicalTrials.gov, NCT05307718) is being conducted in accordance with the Declaration of Helsinki and was approved by the Ethics Committees of the School of Medicine, University of Zagreb, (reg. number 380-59-10106-20-111/125; class 641-01/20-02/01, 29 September 2020) and the University Hospital Centre Zagreb (class 8.1-20/142-2; number 02/21 AG, 7 September 2020), Croatia. Written informed consent for genotyping the pharmacogenes of interest and for publishing anonymized data for scientific purposes was signed by the patient. His cardiac history was notable for two previous myocardial infarctions managed with percutaneous coronary intervention and coronary artery bypass grafting, as well as implantation of a cardioverter–defibrillator. Further pharmacologic optimization was limited due to persistent hypotension. On admission, the patient presented with signs of global heart failure and severely impaired cardiac function, with an ejection fraction of 20% and elevated NT-proBNP (5623 ng/L). Normal range of NT-proBNP by age and gender is <125.0 ng/L. Hemodynamic evaluation revealed severe biventricular dysfunction and pulmonary hypertension. After exhausting all conventional therapeutic options, the patient was indicated for heart transplantation.

Due to pre-sensitization and the presence of donor-specific antibodies (DSA), perioperative immunoadsorption was indicated. An initial series of five procedures was planned, with additional treatments considered if necessary. Early postoperative management also included intravenous immunoglobulins administered as prophylaxis against cytomegalovirus infection.

Within a month of being listed on the Eurotransplant urgent list, the patient underwent successful orthotopic heart transplantation using the bicaval technique. Due to pre-sensitization and the presence of donor-specific antibodies (DSA), perioperative immunoadsorption was indicated. Early postoperative management also included immunoglobulins administered as prophylaxis against cytomegalovirus infection. Mild thrombocytopenia was observed postoperatively. Additionally, persistent pleural drainage was noted, with transient pleural effusions managed conservatively. Echocardiography confirmed preserved graft function (EF 60%), and no signs of rejection were seen on endomyocardial biopsies (ISHLT Grade 0–1R).

The main therapeutic challenge was achieving target trough levels of tacrolimus, introduced on postoperative day six. Despite daily dose escalation, trough levels measured by TDM remained subtherapeutic (3–5 ng/mL) for over a month, prompting pharmacogenetic testing to investigate potential metabolic causes.

Targeted concentrations of tacrolimus in the first month after transplantation are 12–15 ng/mL, 10–15 ng/mL up to 6 months, and then 5–10 ng/mL long-term.

Genomic DNA was extracted from whole blood samples with a QIAamp DNA Blood Mini Kit (Qiagen, Hilden, Germany). Genotyping analysis was performed on an Applied Biosystems 7500 Real-Time PCR System (Applied Biosystems, Waltham, MA, USA) using validated TaqMan SNP Genotyping Assay for CYP3A4*22 (rs35599367; assay ID: C__59013445_10) and TaqMan Drug Metabolism Genotyping Assays for CYP3A4*1B (rs2740574; assay ID: C___1837671_50), CYP3A5*3 (rs776746; assay ID: C__26201809_30).

Pharmacogenetics test results (Table 1) revealed that the patient is a normal CYP3A4 metabolizer (genotype *1/*1) and intermediate metabolizer for CYP3A5 (CYP3A5 expresser, carrying one normal function allele and one no function allele, genotype *1/*3), associated with faster metabolism of tacrolimus. It is estimated that CYP3A5 enzyme expression is present in about 10% to 20% of individuals of European descent [6,7].

The patient’s *CYP3A4* and *CYP3A5* genotypes combination indicated enhanced overall CYP3A-mediated metabolism relative to typical heart transplant recipients, explaining the persistently subtherapeutic tacrolimus levels. According to the guidelines for *CYP3A5* genotype and tacrolimus dosing [3], patients with this genetic profile require an initial tacrolimus dose approximately 1.5 to 2 times higher than the standard initial dose, with the maximum recommended daily dose capped at 0.3 mg/kg/day. For this patient, weighing 98 kg, the calculated maximum recommended daily dose was approximately 29.4 mg.

Initially, the patient was administered tacrolimus at 3.5 mg twice daily, which was subsequently increased to 7 mg twice daily. Additionally, diltiazem, a CYP3A4 inhibitor, was introduced at a dose of 60 mg twice daily to improve tacrolimus bioavailability. Further dose adjustments were guided by TDM. Following these pharmacogenetically informed interventions, tacrolimus concentrations gradually reached the therapeutic target range (12–15 ng/mL during the first month post-transplant, subsequently lowered to 5–10 ng/mL).

At the nine months post-transplantation follow-up, the patient remained asymptomatic with stable graft function, absence of rejection signs, and good tolerability to immunosuppressive therapy.

At discharge, the maintenance immunosuppressive regimen included tacrolimus (6.5 mg twice daily), mycophenolate mofetil (1.5 g twice daily), prednisone (15 mg daily), and diltiazem (90 mg twice daily), along with prophylactic antimicrobial therapy and other required pharmacotherapies. Blood pressure and body weight were stable, with normal appetite and diuresis reported. Additionally, the patient reported excellent exercise tolerance.

### 2.2. Recommendations for Tacrolimus Dosing Based on Pharmacogenetic Data

Several international organizations have issued pharmacogenetically informed guidelines for tacrolimus dosing and recommendations for pre-therapy genotyping. These include the Clinical Pharmacogenetics Implementation Consortium (CPIC) [3], the Dutch Pharmacogenetics Working Group (DPWG) [5], and the French National Network of Pharmacogenetics (RNPGx) [9]. Additional recommendations are also available from the U.S. Food and Drug Administration (FDA) [10]. According to these bodies, genetic variability in CYP3A enzymes, particularly CYP3A5, has a major impact on systemic tacrolimus concentrations and the ability to achieve therapeutic targets in transplant recipients.

The CPIC guidelines emphasize that tacrolimus blood concentrations are significantly influenced by the CYP3A5 genotype, with extensive evidence correlating genotype with phenotypic variability. Over 50 studies have shown that kidney, heart, and lung transplant recipients carrying at least one *CYP3A5* *1 allele (*1/*1 or *1/*3 genotypes, expressers) present significantly lower tacrolimus trough levels at standard doses compared to *3/*3 homozygotes (non-expressers). These subjects, classified as normal or intermediate metabolizers, typically require an increased starting dose, 1.5 to 2 times higher than standard, without exceeding 0.3 mg/kg/day. Close TDM is recommended to minimize the risk of toxicity, such as arterial vasoconstriction, hypertension, and nephrotoxicity, which may occur with supratherapeutic tacrolimus concentrations. The CPIC also notes that rare alleles such as *CYP3A5* *2, *8, and *9 may be encountered, although their functional impact remains uncertain. However, if at least one *1 allele is present, an intermediate metabolizer phenotype should be assumed [3].

The French RNPGx network supports CPIC recommendation and similarly advises increasing the starting dose of tacrolimus by 1.5 to 2 times in carriers of *CYP3A5* *1/*1 or *1/*3, with a maximum of 0.3 mg/kg/day and subsequent adjustment based on TDM. Moreover, the RNPGx recommends pre-transplant genotyping of *CYP3A5* in all kidney, heart, and lung transplant patients before immunosuppressive therapy initiation. While the additional benefit of *CYP3A4* genotyping at therapy initiation still requires further evaluation, retrospective analysis of both *CYP3A5* and *CYP3A4* genotypes may be useful, particularly in the case of atypical tacrolimus pharmacokinetics [9].

The DPWG also endorses genotype-guided dosing, classifying patients as either heterozygous or homozygous *CYP3A5* expressers. A 1.5-fold dose increase is recommended for heterozygous expressers and up to a 2.5-fold increase for homozygous expressers, with dose adjustments guided by TDM. However, the DPWG currently does not recommend routine genotyping in clinical practice because TDM is available [11].

According to the FDA, patients classified as intermediate or normal CYP3A5 metabolizers are at increased risk of subtherapeutic tacrolimus exposure and subsequent graft rejection. This is reflected in the agency’s pharmacogenomic biomarker tables, where such subjects are identified as a genotype-defined subgroup for whom standard dosing may be insufficient [10].

In contrast to these organizations, which provide detailed genotype-based dosing guidance for tacrolimus, the European Medicines Agency (EMA) includes pharmacogenomic information primarily within the Summary of Product Characteristics (SmPC) for approved tacrolimus formulations. While CYP3A-related considerations may be mentioned, EMA SmPCs generally emphasize TDM as the primary approach to dose individualization, without specific recommendations based on CYP3A5 genotype or metabolizer phenotype. Analyses have shown that EMA drug labels often contain limited pharmacogenetic content, and only a subset of actionable gene–drug interactions are systematically covered [12]. For tacrolimus, SmPC recommendations typically suggest initiating therapy at 0.1–0.3 mg/kg/day, with subsequent adjustments based on whole-blood trough concentrations (TDM) and clinical response, without reference to genetic testing [13]. Thus, although the EMA recognizes the relevance of pharmacogenomics, the agency does not currently provide the structured, phenotype-specific dosing algorithms available from other international expert groups.

A summary of tacrolimus dosing recommendations based on the *CYP3A5* genotype is presented in Table 2, comparing guidance from the CPIC, DPWG, and RNPGx. Among them, only the CPIC guidelines have a strong classification of recommendations [3,5,9].

Taken together, these pharmacogenetically driven guidelines consistently support pre-treatment genotyping of *CYP3A5*, and, to a growing extent, *CYP3A4*, particularly in solid organ transplant recipients. Such stratification can improve the likelihood of achieving therapeutic tacrolimus levels early in treatment, thereby enhancing transplant success rates and reducing the need for trial-and-error dose adjustment.

A consensus document developed by the Association for Molecular Pathology (AMP) in collaboration with several major pharmacogenomics organizations outlines unified recommendations for genotyping *CYP3A4* and *CYP3A5* in clinical pharmacogenetic applications [8]. These guidelines emphasize the inclusion of certain high-priority alleles, referred to as Tier 1, based on their clinical significance. These include the CYP3A4*22 allele, which is associated with reduced enzyme function, and the CYP3A5*3, *6, and *7 alleles, all of which result in non-functional proteins [8].

In contrast, Tier 2 alleles, such as CYP3A4*20, are considered supplementary due to their lower population frequencies or more limited supporting evidence. Notably, despite its frequent appearance in scientific literature, the allele is not recommended for standard testing, as it retains normal enzymatic function and lacks proven clinical impact [8].

The allele selection in the guidelines was based on factors such as functional effects, allele frequency across populations (with Tier 1 requiring a minor allele frequency of at least 1%), availability of reference standards, and the practicality of detecting these variants using routine laboratory methods. Although the recommendations do not include phenotype interpretations or dosing advice, they serve to standardize pharmacogenetic testing and emphasize variants that are most relevant for drugs like tacrolimus, which are primarily metabolized by CYP3A enzymes [8].

A recently reported clinical case highlights the importance of performing CYP3A genotyping prior to kidney transplantation. In this instance, a patient developed acute cellular rejection (ACR) on the eighth day following transplantation, during a period in which tacrolimus blood levels were consistently subtherapeutic. This outcome emphasizes how pharmacogenetic testing can support more accurate initial dosing of tacrolimus, helping to maintain therapeutic levels and potentially reducing the risk of transplant rejection [14]. Although CPIC guidelines for CYP3A5 genotype-based tacrolimus dosing have been available since 2015, their implementation in clinical practice, particularly in transplant centers, has remained limited. The case also demonstrates the potential value of integrating additional pharmacogenetic markers, including variants in *CYP3A4* and transporter genes, into dosing strategies. Specifically, the patient was identified as a *CYP3A5* expresser and a carrier of the CYP3A4*1B allele, both of which are, according to some but not all authors [8], associated with increased enzymatic activity and enhanced tacrolimus metabolism, likely contributing to the observed rapid drug clearance [7]. According Pratt et al., the CYP3A4*1B allele is associated with normal function and is, therefore, not included in the Tier 1 or 2 recommendations for routine clinical testing [8].

## 3. Discussion

Tacrolimus pharmacogenetics have been extensively studied, with the majority of research focusing on polymorphisms in *CYP3A5*, *CYP3A4*, and *ABCB1* genes, given their central role in the drug’s metabolism and transport [1,4]. Among these, the CYP3A5*3 allele (rs776746) has shown the most robust and reproducible association with tacrolimus dose requirements, particularly in individuals of European ancestry, where the non-expressor *3/*3 genotype predominates [6,15,16].

The CYP3A4*22 (rs35599367) allele has been associated with reduced CYP3A4 expression and decreased enzymatic activity [8]. The clinical implications of the CYP3A4*1B (rs2740574) variant remain unclear, as ongoing research continues to evaluate its potential role in upregulating gene expression and enhancing CYP3A4 enzyme activity [7]. On the other hand, individuals who carry at least one CYP3A5*1 allele, whether heterozygous or homozygous, are classified as CYP3A5 expressers and typically require increased tacrolimus dosages to achieve therapeutic drug levels. According to current CPIC/PharmGKB data, *CYP3A5* expressers (normal and intermediate metabolizers) represent approximately 14.2% of individuals of European descent, with the majority (85.7%) being poor metabolizers (non-expressers) [6].

According to Elens et al., considering CYP3A4*22 and CYP3A5*3 alleles, frequencies of poor, intermediate, and normal metabolizers in Caucasian (8%, 74%, 18%), Asian (-, 50%, 50%), and African (-, 10%, 90%) populations are shown in Table 3 [17].

The 2021 study by Déri et al. investigated the concept of CYP3A status, defined as the combined influence of the *CYP3A5* genotype and CYP3A4 expression on tacrolimus pharmacokinetics in heart transplant recipients [18]. A notable finding of this study was the observation of linkage disequilibrium (LD) between *CYP3A* alleles, specifically between CYP3A4*1B and CYP3A5*1 (rs2740574 and rs776746, respectively), suggesting co-inheritance due to their proximity on the chromosome [18]. The study confirmed that CYP3A5 expressers (individuals carrying at least one *1 allele) required approximately 2.4 times higher tacrolimus doses to reach therapeutic concentrations compared to non-expressers (*3/*3). Furthermore, among non-expressers, those with low CYP3A4 expression (measured by mRNA in leukocytes) exhibited the highest dose-adjusted tacrolimus concentrations, indicating markedly slower metabolism. These findings emphasize the cumulative impact of both CYP3A5 and CYP3A4 activity on tacrolimus disposition [18].

The same study also reported that methylprednisolone, used during early post-transplant immunosuppression, upregulated CYP3A4 expression, which declined over time as steroid doses were tapered. Interestingly, tacrolimus clearance remained relatively stable in CYP3A5 expressers but fluctuated significantly in non-expressers depending on CYP3A4 expression dynamics. These results support a genotype-informed dosing strategy based on combined CYP3A status, allowing earlier identification of patients likely to require significant dose adjustments, thereby potentially improving clinical outcomes and minimizing toxicity [18]. A separate study by Hannachi et al. examined the distribution of three CYP3A polymorphisms—CYP3A4*1B, CYP3A4*22, and CYP3A5*3—in a Tunisian population that included 101 healthy individuals and 102 kidney transplant recipients [19]. The most prevalent genotype combination was CYP3A4*1/*1 and CYP3A5*3/*3, identified in 60.6% of participants.

No significant differences in genotype frequencies were observed between the healthy and transplant groups, suggesting that these variants are not disease-specific and may be applicable for broader pharmacogenetic implementation. Additionally, the allele and genotype frequencies observed in the Tunisian cohort closely mirrored those reported in Caucasian populations, supporting the external generalizability of CYP3A pharmacogenetic data [19].

The recent genome-wide study by Richard-St-Hilaire et al. (2024) provides valuable evolutionary and regulatory insight into the debated role of the CYP3A4*1B variant [20]. Their findings confirm strong signatures of positive selection on CYP3A5 and CYP3A4, particularly in African populations, and reveal instances of unusual linkage disequilibrium (uLD) within the *CYP3A* gene cluster. Notably, a strong uLD signal was observed between *CYP3A5* and *CYP3A43*, a gene previously underexplored in pharmacogenetics. Variants in *CYP3A43* were identified as expression quantitative trait loci (eQTLs) for CYP3A5, suggesting possible co-regulation mechanisms that could influence interindividual variability in drug response. Through Mendelian randomization, the authors also demonstrated a causal relationship between CYP3A5 eQTLs and reticulocyte counts, proposing a possible evolutionary advantage—such as malaria resistance—that may explain the prevalence of expression alleles in African populations. These insights underscore the importance of interpreting pharmacogenetic markers within their broader evolutionary and population-specific genetic contexts, especially since current clinical guidelines, such as those from the CPIC, are largely based on data from European cohorts [20].

Two recent studies by Deininger et al. (2016) and Hernandez et al. (2024) examined the influence of *CYP3A* genetic polymorphisms, particularly in *CYP3A5* and *CYP3A4*, on tacrolimus pharmacokinetics in adult heart transplant recipients [21,22]. Both studies confirmed the established association between CYP3A5*3 and reduced tacrolimus clearance, with expressers requiring higher doses to reach target trough levels. However, they diverged in their assessment of *CYP3A4* variants.

Deininger et al. found that CYP3A4*22 had no significant effect on tacrolimus pharmacokinetics when considered alone or in combination with CYP3A5*3. Their data from stable, long-term post-transplant patients suggested that routine *CYP3A4* genotyping may offer limited additional predictive value beyond CYP3A5 status alone [21]. In contrast, Hernandez et al., studying patients in the early post-transplant period, demonstrated that combined CYP3A phenotypes—including *1B, *1G, and *22—accounted for up to 52% of variability in tacrolimus exposure, particularly among CYP3A5 non-expressers [22].

These contrasting findings likely reflect differences in the clinical time points examined: the early phase of immunosuppression, characterized by dynamic physiological changes and phenoconversion, versus the more stable maintenance phase. Hernandez et al. suggest that comprehensive *CYP3A4* and *CYP3A5* genotyping may be particularly valuable in the early post-transplant period to guide initial dosing, while *CYP3A5* genotyping alone may suffice during long-term follow-up.

Adding a different perspective, the study by Concha et al. (2024) investigated the independent contribution of CYP3A4*1B in a cohort of Spanish solid organ transplant recipients [23]. They found that carriers of CYP3A4*1B required higher tacrolimus doses and had lower dose-adjusted trough concentrations, regardless of CYP3A5 status. Furthermore, a correlation was reported between CYP3A4*1B and PXR 69789GG genotypes, suggesting a potential combined effect on hepatic metabolic capacity. Although the study’s sample size was limited, these findings point to a possibly stronger and independent role of CYP3A4*1B in tacrolimus pharmacokinetics.

Despite extensive pharmacogenetic investigation, only CYP3A5*3 has consistently demonstrated a strong and clinically actionable association with tacrolimus pharmacokinetics. In contrast, evidence regarding *CYP3A4* and other loci remains heterogeneous. Study outcome variability may be attributed to ethnic differences, small sample sizes, inconsistent pharmacokinetic assays, timing of measurement, and donor genotype influence. Further large-scale, multi-ethnic studies and meta-analyses that incorporate these factors are needed to fully clarify the pharmacogenetic landscape of tacrolimus.

Studies exploring *ABCB1* variants such as 3435C>T (rs1045642) and 2677G>T/A (rs2032582), which affect intestinal expression of P-glycoprotein (Multi-Drug Resistance 1 gene-*MDR1*), an efflux transporter, have yielded inconsistent results regarding their influence on tacrolimus absorption and systemic exposure [24,25]. Additional genes of interest include *NR1I2*, which encodes the pregnane X receptor (PXR), a key regulator of CYP3A and ABCB1 transcription. Several studies have reported associations between *NR1I2* single nucleotide polymorphisms (SNP) and variability in tacrolimus pharmacokinetics, although findings remain preliminary [26]. In 2013, the first data on the influence of ABCC2 variants on tacrolimus pharmacokinetics were reported. ABCC2 encodes Multidrug Resistance-associated Protein 2 (MRP2), a member of the ABC transporter superfamily involved in biliary excretion in hepatocytes [27]. Early studies on *ABCC2* polymorphisms produced inconsistent results, similar to findings for *ABCB1* [24,28]. However, more recent evidence from 2022 clearly demonstrates that the *ABCC2* 1249G>A polymorphism in donors significantly affects tacrolimus dose-adjusted concentrations after a switch to once-daily dosing. This highlights the importance of considering both donor and recipient genotypes when individualizing tacrolimus therapy [29]. Another gene of interest is *SLCO1B1*, which encodes the hepatic uptake transporter OATPB1, also implicated in tacrolimus biliary elimination. One study reported an association between the *SLCO1B1* rs2306283 polymorphism and tacrolimus blood levels [30], while a more recent study suggested that *SLCO1B1* rs2291075 may be a novel variant linked to tacrolimus metabolism and transport [31]. Other candidate genes include *POR* (cytochrome P450 oxidoreductase), which provides electrons to CYP enzymes and may influence tacrolimus metabolism [32,33]. Polymorphisms in *TGFB1*, *PPIA*, and *CYP2C8* have also been proposed as modulators of tacrolimus response, although their clinical relevance in pharmacokinetics or pharmacodynamics remains limited [34,35]. Genetic variants associated with tacrolimus pharmacokinetics and pharmacodynamics are shown in detail in Table 4.

### 3.1. Adverse Drug Reaction and Cost-Effectiveness Considerations

In the PREPARE study (Pre-emptive Pharmacogenomic Testing for Preventing Adverse Drug Reactions), published in 2023 and involving 7000 adult participants, it was demonstrated that preemptive pharmacogenomic testing to guide drug dosing reduced the incidence of adverse drug reactions (ADRs) by approximately 30% compared to conventional dosing based on clinical indicators. The genotyping panel included 50 variants across 12 genes involved in drug metabolism, selected based on recommendations from the DPWG [37].

Additional findings from the study showed that 94% of participants carried at least one clinically actionable pharmacogenetic variant, and many individuals carried two or three such variants. In total, 25% of patients had a relevant genetic variant affecting the medication prescribed [37].

Overall, the most frequently prescribed pharmacogenetically relevant drugs were atorvastatin, clopidogrel, and tacrolimus. The implementation rate of DPWG recommendations based on pharmacogenetic test results was approximately 70% [37].

In a recent cost-effectiveness analysis, Deininger et al. evaluated the economic and clinical impact of *CYP3A5* genotype-guided tacrolimus dosing compared to standard-of-care (SOC) dosing across kidney, liver, heart, and lung transplant recipients from the perspective of the U.S. healthcare payer system. Using decision tree models, the study assessed outcomes over the first six months post-transplantation, incorporating genotype frequencies, real-world tacrolimus exposure data (measured as TAC time in therapeutic range using the Rosendaal algorithm), clinical event rates (acute nephrotoxicity, acute cellular rejection, mortality), and cost data derived from Medicare Fee Schedules and published literature [38].

The study reported incremental costs per avoided adverse event for genotype-guided versus SOC dosing as follows: USD 176,667 (kidney), USD 364,000 (liver), USD 12,982 (heart), and USD 93,333 (lung). The probability of achieving overall cost savings with genotype-guided dosing varied across organ types, with the highest likelihood observed in heart (51.8%) and lung (54.1%) transplant recipients [38].

Key drivers of cost-effectiveness included the proportion of patients maintaining high tacrolimus time-in-therapeutic range (TTR) and physician uptake of genotype information into clinical decision making. Overall, the genotype-guided strategy demonstrated modest clinical benefits at increased cost relative to SOC, but may be justified in populations with a high prevalence of *CYP3A5* expressers. The authors underscored the need for further economic evaluations focusing on intermediate outcomes, such as dose adjustments, especially in high-risk subgroups [38].

Complementing this analysis, findings from the eMERGE-PGx project, funded by the National Institutes of Health (NIH), underscore the broader economic potential of preemptive pharmacogenomic testing [39]. By integrating genotyping into electronic health records across multiple healthcare systems, the project evaluated both clinical utility and financial feasibility of early genetic testing. A decision-analytic model within this initiative, referred to as the “Test All” strategy, simulated the use of a hypothetical pharmacogenomic panel to guide initial therapy selection [40]. Assuming a 15% prevalence of actionable variants, a test cost of USD 200, and respective efficacies of 74% and 60% for first- and second-line therapies, the model projected per-patient cost savings between USD 200 and USD 767 [40].

These savings were most pronounced in scenarios where pharmacogenomic information could prevent inappropriate therapy selection, particularly in patients with high-prevalence variants. While initial testing incurs upfront costs, the long-term economic impact favors testing when considering the avoidance of ADRs, improved treatment efficacy, and reduction in trial-and-error prescribing. The main cost drivers identified were test cost and the cost burden of ineffective therapies, reinforcing that in populations with common genotypes, universal testing may be not only clinically valuable but also cost effective or cost neutral [38,39,40].

### 3.2. Drug Interactions

Healthcare professionals involved in the care of transplant recipients must remain vigilant about the potential for drug–drug interactions (DDIs), particularly when medications are added to or withdrawn from the therapeutic regimen. The most common scenarios requiring close monitoring of adverse effects and immunosuppressant concentrations occur following the initiation of anti-infective therapy [41].

Although most DDIs are not strictly contraindicated, many can lead to serious outcomes—such as allograft rejection or acute kidney injury—if left unmanaged. In this context, medication reviews and pharmacotherapy assessments by clinical pharmacists can provide valuable support [41].

### 3.3. Pharmacokinetic Interactions

Pharmacokinetic DDIs typically occur when co-administered drugs alter tacrolimus blood levels by affecting absorption, metabolism, or elimination. CNIs and PSIs are metabolized predominantly by CYP3A4, making them susceptible to enzyme modulators:

- CYP3A4 inducers (e.g., rifampin, carbamazepine, efavirenz) accelerate metabolism, reducing tacrolimus levels.
- CYP3A4 inhibitors (e.g., macrolides, azoles, diltiazem) reduce metabolism, increasing drug exposure and toxicity risk [42].

### 3.4. Pharmacodynamic Interactions

These occur when co-administered drugs enhance or counteract the pharmacological effects of immunosuppressants:

- Additive myelotoxicity: ganciclovir, valganciclovir, or TMP-SMX can potentiate bone marrow suppression.
- Additive nephrotoxicity: amphotericin B, aminoglycosides, or NSAIDs used with CNIs may elevate nephrotoxic risk [42].

### 3.5. Clinical Pharmacokinetic Modulation with Diltiazem

A 2020 retrospective study showed that adding diltiazem reduced tacrolimus doses by ~52% and monthly costs by ~50% in heart transplant recipients, without compromising efficacy [43]. This effect is due to diltiazem’s inhibition of CYP3A and drug transporter P-glycoprotein (P-gp), slowing tacrolimus metabolism. P-gp is an ATP-dependent transporter protein coded by the *MDR1/ABCB1* gene. It is found in cell membranes and pumps a wide range of drugs and other substances out of cells.

A related RCT in kidney recipients confirmed that CYP3A5 expressers benefit most from low-dose diltiazem, showing improved tacrolimus exposure and reduced dose requirements. Importantly, no significant changes in blood pressure were observed, confirming tolerability [44].

### 3.6. DDI and Pharmacogenetics in Clinical Practice

A case report by Concha et al. illustrates how CYP3A5*3/*3 status combined with the *ABCB1* TTT haplotype and a *PXR* variant (69789A>G) contributed to tacrolimus toxicity in a renal transplant patient. Co-treatment with omeprazole (a CYP3A4 and P-gp inhibitor) further exacerbated adverse events. Switching to rabeprazole resolved toxicity, underscoring the need to consider broader pharmacogenetic profiles beyond *CYP3A5* [45].

### 3.7. Antifungal Interactions: Caspofungin and Clotrimazole

A study in Chinese kidney recipients found that caspofungin reduced tacrolimus exposure by ~11% only in CYP3A5*3/*3 patients, suggesting genotype-specific DDI risks [46]. Similarly, clotrimazole withdrawal in Japanese heart recipients led to a 3.3-fold increase in tacrolimus clearance in expressers vs. 2.2-fold in non-expressers, showing that CYP3A5 status modulates the extent of CYP3A4 inhibition [47].

### 3.8. Atorvastatin and Shared CYP3A Pathways

Atorvastatin is frequently co-prescribed in heart transplant recipients. A study in Egyptians reported that carriers of CYP3A4*1B (T/T) and CYP3A5*3 (C/C) had greater triglyceride reductions, but also higher atorvastatin plasma levels, prolonged half-life, and increased ALT, AST, and CK levels. Since both tacrolimus and atorvastatin share CYP3A metabolism, co-administration warrants attention to avoid toxicity [48].

## 4. Conclusions

This case and the accompanying review underscore the growing clinical utility of pharmacogenetics in optimizing tacrolimus therapy after heart transplantation. While therapeutic drug monitoring (TDM) remains the cornerstone of immunosuppressive management, integrating pharmacogenetic testing—particularly for CYP3A5 and CYP3A4 variants—enhances predictive accuracy, especially in patients with unexplained variability in drug exposure.

The presented patient’s CYP3A5 expresser status, combined with normal CYP3A4 activity, accounted for subtherapeutic tacrolimus concentrations despite standard dosing. A genotype-informed strategy, including dose escalation and adjunct therapy with a CYP3A4 inhibitor, enabled achievement of target levels and contributed to successful clinical outcomes.

Beyond this case, extensive evidence supports routine incorporation of pharmacogenetic profiling into transplant care. The CYP3A5*3 allele remains the most robust predictor of tacrolimus dose requirements, while *CYP3A4* variants—particularly *22 and *1B—appear to exert context-dependent effects, particularly during early or unstable therapy phases. Gene–gene interactions and linkage disequilibrium (e.g., between CYP3A4*1B and CYP3A5*1) further complicate metabolism prediction.

Drug–drug interactions add an additional layer of complexity, with agents like diltiazem, clotrimazole, caspofungin, and statins modulating tacrolimus exposure based on the *CYP3A* genotype. Evidence from pharmacokinetic studies and real-world data suggests that pharmacogenetic testing can also inform DDI management and support cost-effective therapeutic strategies.

Altogether, this integrative approach—combining pharmacogenetics, TDM, and DDI awareness—enables more precise, individualized immunosuppression. Widespread implementation of genotype-guided protocols could improve outcomes and resource utilization, particularly in high-risk or pharmacokinetically unpredictable patients.

## Figures and Tables

**Table 1:** Pharmacogenetic profile of the patient.

Gene/Alleles Tested	Genotype	Phenotype	Interpretation
*CYP3A4* *1B, *22	*1/*1	Normal Metabolizer (NM)	Normal metabolizer of CYP3A4 drug substrates, including immunosuppressants.
*CYP3A5* *3	*1/*3	Intermediate Metabolizer (IM)—Expresser	Intermediate expression/activity of the CYP3A5 enzyme, suggesting a faster metabolism of CYP3A5 drug substrates compared to the common lack of CYP3A5 enzyme activity in the general population (carriers of non-functional *CYP3A5* *3/*3 alleles—non-expressers).

CYP3A interpretation: Based on this combination of CYP3A4 and CYP3A5 genotypes, the patient is classified as a normal CYP3A metabolizer, meaning a faster metabolism of CYP3A drug substrates compared to individuals with the commonly observed intermediate CYP3A metabolizer phenotype. Non testing for alleles CYP3A5*6 and *7 is a limitation of this study.

**Table 2:** Comparison of genotype-based tacrolimus dosing recommendations [3,5,9].

	CYP3A5 Phenotype	*CYP3A5* Genotype	Recommended Initial Dose Adjustment	Notes
	NM (homozygous expressor)	*1/*1	1.5–2× standard dose (max 0.3 mg/kg/day)	Formal recommendation; adjust based on TDM
CPIC	IM (heterozygous expressor)	*1/*3, *1/*6, *1/*7	1.5–2× standard dose (max 0.3 mg/kg/day)	Formal recommendation; adjust based on TDM; rare alleles noted (*6, *7)
	Non-expressers	*3/*3, *6/*6, *7/*7, *3/*6, *3/*7, *6/*7	Standard starting dose	Formal recommendation; adjust based on TDM;
DPWG	Homozygous expresser	*1/*1	2.5× dose (max 0.3 mg/kg/day)	Adjust using TDM
	Heterozygous expresser	*1/*3, *1/*6, *1/*7	1.75× standard dose (max 0.3 mg/kg/day)	Adjust using TDM
	Non-expresser	*3/*3, *6/*6, *7/*7, *3/*6, *3/*7, *6/*7	Standard dose	Adjust using TDM
	Expressers (intermediate/normal)	*1/*3, *1/*1, *1/*6, *1/*7	1.5–2× dose (max 0.3 mg/kg/day)	Advisable level Pre-transplantation pharmacogenetic tests
RNPGx	Non-expressers	*3/*3, *6/*6, *7/*7, *3/*6, *3/*7, *6/*7	Standard dose	-

CPIC—Clinical Pharmacogenetics Implementation Consortium, DPWG—Dutch Pharmacogenetics Working Group, RNPGx—French National Network of Pharmacogenetics, TDM—therapeutic drug monitoring.

**Table 3:** Frequencies of CYP3A predicted phenotype in several population based on combined CYP3A4*22 and CYP3A5*3 genotype [17].

CYP3A Phenotype	CYP3A4*22 Genotype	CYP3A5*3 Genotype	Caucasian	Asian	African
Poor metabolizer	*1/*22	*3/*3	8	-	-
	*22/*22	*3/*3			
Intermediate metabolizer	*1/*1	*3/*3	72	50	10
	*1/*22	*1/*3			
	*1/*22	*1/*1			
	*22/*22	*1/*1			
Normal metabolizer	*1/*1	*1/*1	18	50	90
	*1/*1	*1/*3			

**Table 4:** Genetic variants associated with tacrolimus pharmacokinetics and pharmacodynamics.

Gene	Variant(s)	rsID(s)	Functional Effect/Role	Impact on Tacrolimus Levels	Strength of Evidence	Key References
*CYP3A5*	*3	rs776746	Non-functional allele, reduced metabolism	Higher levels in *3/*3 (non-expressers)	Strong, consistent	[1,3]
*CYP3A4*	*22	rs35599367	Reduced gene expression and enzymatic activity	Higher levels due to reduced metabolism	Moderate, emerging	[6,14]
*CYP3A4*	*1B	rs2740574	Possible increased activity, linkage with CYP3A5*1	Lower levels in some populations	Limited, population-specific	[5,25,36]
*ABCB1*	C3435T, G2677T/A	rs1045642, rs2032582	Altered P-gp expression and activity, inconsistent findings	Variable absorption; mixed effect on levels	Conflicting	[25,26,27]
*ABCC2*	c.-24C>T	rs717620	Affects expression of MRP2 transporter	Possible altered biliary elimination	Limited, inconsistent	[28,29]
*SLCO1B1*	rs2306283, rs2291075	rs2306283, rs2291075	Involved in hepatic uptake (OATP1B1), potential PK effects	Some variants associated with higher levels	Preliminary	[30,31]
*NR1I2* (PXR)	69789A>G	rs2472677	Regulates CYP3A and ABCB1 transcription	Indirect effect via altered enzyme expression	Preliminary	[28]
*POR*	A503V	rs1057868	Electron donor for CYP enzymes, may influence metabolism	Possible effect on CYP3A enzyme activity	Limited	[32]
*CYP2C8*	*3	rs10509681, rs11572080	Involved in arachidonic acid metabolism (PD effects)	Hypothesized role; no direct PK data	Hypothesis-generating	[34]
*TGFB1*, *PPIA*	c.-509C>T, rs6850	rs1800469, rs6850	Inflammatory/immune modulation pathways	Under investigation	Under investigation	[34,35]

## Data Availability

The original contributions presented in this study are included in the article. Further inquiries can be directed to the corresponding author.
